# Participants’ Experiences of a Workplace-Oriented Problem Gambling Prevention Program for Managers and HR Officers: A Qualitative Study

**DOI:** 10.3389/fpsyg.2019.01494

**Published:** 2019-07-03

**Authors:** Jonas Rafi, Ekaterina Ivanova, Alexander Rozental, Petra Lindfors, Per Carlbring

**Affiliations:** ^1^Department of Psychology, Stockholm University, Stockholm, Sweden; ^2^Department of Clinical Neuroscience, Karolinska Institutet, Stockholm, Sweden; ^3^Great Ormond Street Institute of Child Health, University College London, London, United Kingdom; ^4^Department of Psychology, University of Southern Denmark, Odense, Denmark

**Keywords:** health promotion, problem gambling, workplace intervention, harmful use, prevention, qualitative content analysis

## Abstract

Workplace health promotion programs (WHPPs) refer to a set of health promotion and protection strategies implemented at a worksite and designed to meet the health and safety needs of employees. One important question for WHPPs is how middle management experience their participation in a WHPP. This study aims to explore this question further by applying a qualitative content analysis to interviews with thirteen managers and ten human resource officers participating in a WHPP focusing on problem gambling. The WHPP consisted of two components: policy implementation and skills-development training. The participants were interviewed about their experiences of these two components and the implementation process. The qualitative content analysis resulted in six themes: (1) Expectations of the skills-development training, (2) Experiences of and prior beliefs about problem gambling, (3) A good foundation, (4) The difficult conversation, (5) Appreciated aspects of the training sessions, and (6) Remaining obstacles. The results suggest that the presentation of cases, facts, and general knowledge was appreciated by most participants. However, participants also expressed that they would benefit from tailored interventions, more support in the policy implementation process, and following up on the results.

## Introduction

The workplace is an important area for health promotion, considering the possibility of reaching a large population there and the amount of time spent at work. Workplace health promotion programs (WHPPs) exist for a variety of issues (Wild et al., 2006) and are considered to be important tools for contributing to good public health (Magnavita et al., 2017). Previous research has focused on both effects of specific interventions (e.g., Bruening et al., 2015; Risica et al., 2018) and factors relating to the implementation of WHPPs in general. Examples of the latter include employee and employer opinions (McCleary et al., 2017) and needs (Kilpatrick et al., 2014) related to WHPPs. Research on the effectiveness of WHPPs has yielded mixed results, depending on the program and target (Buchberger et al., 2011; Goetzel et al., 2014; Hendriksen et al., 2016; Ryu et al., 2017). Furthermore, the quality of the interventions and their reporting varies, and there are unresolved issues regarding what makes WHPPs effective and how the programs are perceived by employees. Previous research shows that managers play an important role in successful implementation of WHPP and that middle management need more knowledge and support in this area (Larsson et al., 2016; Wiman et al., 2016; Justesen et al., 2017).

Despite being endorsed by many (e.g., Griffiths, 2009; Binde, 2016b), few WHPPs target problem gambling and the effects of implementing a WHPP targeting problem gambling (PG) prevention remains to be explored. PG is defined as “excessive gambling behavior that creates negative consequences for the gambler, others in his/her social network, and for the community” (Wynne and Ferris, 2001, p. 8). Interventions for preventing and treating PG come in different forms, from telephone counseling (Rodda and Lubman, 2012) to treatment protocols based on cognitive behavioral therapy (Casey et al., 2017). However, to our knowledge there are no interventions aimed specifically toward workplace prevention of PG. PG remains a public health problem in many countries (Marshall, 2009), and preventing PG is thus warranted. In Sweden, the estimated problem gambling prevalence is 1.7% (Public Health Agency of Sweden, 2016b), and 18% of the adult Swedish population are concerned about the gambling habits of a significant other (Svensson et al., 2013; Magnusson et al., 2015).

The idea that the workplace is a suitable venue for the prevention of PG is backed by earlier research showing that 70% of callers to a PG helpline are currently employed (Hawley et al., 2007) and approximately 3% of the working population in Sweden has, at least once during the last year, neglected work or studies in order to gamble (Public Health Agency of Sweden, 2016a). Furthermore, Wood and Williams (2007) noted that 4.3% of a sample of Internet gamblers used a computer located at the workplace as their primary gambling computer, indicating widespread use of gambling in the workplace. In the nearby field of alcohol abuse, a systematic review of workplace interventions for alcohol-related harms (Webb et al., 2009) found evidence for potential benefits of brief interventions, psychosocial skills training, and peer referral. Suggestions for workplace-related interventions for PG include implementing gambling policies, raising awareness of PG, attention to signs of gambling-related harms, control functions, rehabilitation, and appropriate responses to harmful gambling (Binde, 2016b). For alcohol and other drugs (AOD), workplace policies on AOD use have been shown to be associated with less AOD use both at and away from the workplace (Pidd et al., 2016). Although there are differences between gambling and AOD, the effects of using workplace controls (e.g., policies, levels of supervision) are likely to be comparable for PG and other types of harmful use. However, the relationship between workplace policies and PG still needs to be explored.

Even though no workplace intervention for PG has been evaluated, some non-applied research on PG related to the workplace has been conducted (e.g., Griffiths, 2009; Binde, 2016a). One notable contribution is the outline of categories of gambling-related harms published by Langham et al. (2016). Among the workplace-related harms associated with PG are reduced performance, increased absenteeism, job loss, fraud, theft, and reduced opportunity for employment due to poor past performance or criminal activity (Langham et al., 2016). Given the occurrence of gambling in the workplace and its potential work-related harms, the workplace can be considered an appropriate venue for detecting problem gambling. The current intervention is timely given recent suggestions that the prevention paradox framework is applicable to problem gambling (Canale et al., 2016). The prevention paradox states that by focusing on a large population with low risk for a given condition, larger preventive effects can be attained compared to aiming an intervention at a small population of high-risk individuals. Although evidence for the prevention paradox exists for alcohol (Spurling and Vinson, 2005), there is an ongoing debate of the prevention paradox with regard to PG. Some (Delfabbro and King, 2017) warn that the prevention paradox is not generalizable to the field of PG, while others claim evidence for its presence (Browne and Rockloff, 2018).

To our knowledge, no study has investigated the experiences of participants in a WHPP for PG. The current study aims to fill this gap by applying qualitative content analysis to interviews with managers and human resource officers (HR officers) in mid-to-large-sized organizations who have participated in a WHPP targeting PG.

## Materials and Methods

### Design

This study was part of a cluster-randomized controlled trial investigating the potential effects of a workplace PG prevention program. Ten organizations were randomized to either an intervention group or a waitlist group. The five organizations in the intervention group received the intervention between October 2016 and February 2017. The participating organizations differed in their context: administrative, educational, and manual labor. For the complete description of the cluster-randomized trial, see the published study protocol (Rafi et al., 2017). The qualitative interviews with the five organizations from the intervention group were carried out during spring 2017.

### Intervention

The prevention program and its components were created and delivered by a non-profit organization providing workplace interventions focused on harmful use (e.g., alcohol, drug, and gambling services). The WHPP consisted of two main components: (1) policy development and implementation and (2) skills-development training. The policy component aimed to promote clear roles and responsibilities for managers and HR officers regarding employee problems, and the skills-development component aimed to increase early workplace interventions directed toward individuals showing signs of developing or having developed harmful use. This can be contrasted to WHPPs that aim to promote behavior change at the employee-level. Early interventions included mapping out a potential problem as well as a follow-up including an action plan, with both these being considered core components to promote wellness and prevent mental illness in various settings (Vera, 2013). Well-defined responsibility areas are a crucial aspect of any successful workplace program implementation (Durlak and DuPre, 2008). The program was delivered by five consultants, one at each workplace. The consultants were trained by the organization providing the intervention and they have between 3 and 7 years of experience of such consulting.

The policy component aimed to help workplaces to implement or update policies for PG and other harmful use. The work on policies was conducted by the consultants in collaboration with HR officers from each workplace and guided by a template for work on policies (Alna, 2015) that was included in the WHPP. The skills-development training was delivered in a group format to HR officers and managers at each workplace. Training was split into two sessions, each approximately 3.5 h. Topics included information on PG, prevalence statistics, risk factors, signs of harmful use, harmful use in general, conversation as a tool of early intervention, roles and responsibilities, role-playing, and case discussions. The training had two main aims related to PG and other harmful use: to improve general knowledge of PG and to increase the participants’ inclination to engage in conversations with employees suspected to have a problem with gambling or other harmful use. At the end of the second session, a checklist for managers and HR officers regarding PG was introduced (Nyqvist, 2015). The checklist aimed to remind and guide participants who attended the training regarding how to act if they were suspecting that an employee or a colleague was having gambling problems or other harmful use issues.

### Participants

The interviewees were recruited during the skills-development sessions. At each session, the leader informed the participants about the possibility to participate in an interview about their experiences of participating in the project and provided them with details on how to sign up if they were interested. A total of 340 managers and HR officers attended the sessions. Thirty-four managers and ten HR officers signed up for the interviews. Those who signed up were contacted by the interviewers by telephone in the order they had signed up. If a participant did not answer, the interviewers continued with the next person on the list. This procedure was repeated until all ten HR officers and at least ten managers were successfully contacted. Twenty-three participants (13 managers and 10 HR officers) were interviewed. Ideally, participants should be recruited iteratively to reach sample saturation (Drisko and Maschi, 2015), but due to time constraints and with only 10 HR officers signing up it was decided to limit the number of managers to minimum 10 as well. The number of employees per manager varied between 9 and 70 employees. Of the 23 interviewees, 17 (74%) were women. Of the 13 managers, four worked in an administrative workplace setting, four within education, and five with manual labor. Nine were women and four were men. The corresponding figures for the HR officers are two in an administrative workplace setting, five within education, and three with manual labor. Eight of the interviewed HR officers were women and two were men. Interviewees received no compensation for participating.

### Data Collection

Semi-structured interviews were carried out by three students as part of their bachelor-level theses in behavioral science. The three interviewers, together with authors AR and PC, developed an interview guide. The interview guide was used to ensure that all participants had the opportunity to elaborate on the same topics (e.g., “Why did you choose to attend the skills-development training?”), regardless of who was conducting the interview. The interview guide was pilot-tested on two HR officers and two managers. Since the interview guide was considered adequate, these interviews were included in the data analysis. The final interview guide covered six sections: (1) an introduction, (2) the organization’s early work with PG, (3) policy implementation and the template, (4) skills-training, (5) the checklist, and (6) other thoughts or questions regarding the project (see Supplementary File 1 for an English translation of the interview guide). The interviews lasted between 25 and 63 min and were conducted during work hours at the interviewees’ workplace. A quiet and neutral location was used for each interview. The time between the skills-development training and the interviews varied between 2 and 4 months.

Before conducting the interviews, the interviewers were trained in interview methodology during a 5-week course which formed part of their studies. Prior to the interview, the interviewees were given a rationale for the interview and information about the procedures for ensuring confidentiality. This included avoiding the publication of any personal information and only publishing material and quotes that cannot be traced back to any specific person. Then, participants were informed that they could opt out from the study at any time without any need to explain why. Finally, the participants signed a written informed consent form declaring that they volunteered participation. All participants volunteered participation. Each interview was recorded and transcribed verbatim by a professional transcriber. Then the recordings were erased, and only the transcribed material was used for coding.

### Data Analysis

The data were analyzed by the three first authors using qualitative content analysis. One interview was randomly selected to be pilot-coded by all three coders. Afterward, the pilot-coding was compared and discussed among the coders, to ensure consensus on how to proceed with the coding of remaining material. The coding was similar for all three coders, and so no further pilot coding was performed. Initially, the method of choice stated in the study protocol was thematic analysis. However, during the discussion around the pilot-coding, it was decided to use qualitative content analysis instead. The rationale for this was that the data were perceived as more useful in terms of its manifest content rather than a combination of a manifest and latent content (Vaismoradi et al., 2013). In practice, focusing on the manifest content means less abstraction and interpretation when coding the collected data. Otherwise, the two methods are comparable in using inductive reasoning. Qualitative content analysis can be used to examine patterns and concepts in a data set. A common approach to examining patterns with qualitative content analysis was described by Elo and Kyngäs (2008). First, the data are prepared by selecting the unit of analysis, which in this study corresponded to an expressed statement of unspecified length. Then, the researchers read through the material several times to familiarize with the data. Then the data are to be organized with codes. This is done by describing the data content, which results in a set of codes. The codes are then grouped into distinct themes that capture their similarities while distinguishing them from other themes (see below for an example of how the process was performed in this study).

**FIGURE 1:**
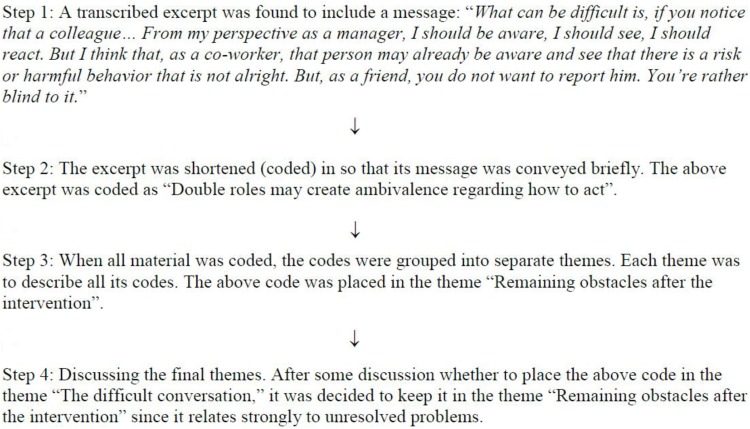
Example of the coding procedure.

After the material was transcribed, author JR divided the material among the coders (JR, EI, and AR). Due to the skewed proportions of managers and HR officers as well as the low sample size, the material was shared sequentially (instead of randomization) to ensure that all coders received similar numbers of HR and manager interviews. The allocation procedure was as follows: the manager interviews were distributed first, with each coder given one interview at a time until all the interviews were handed out. The same procedure was used for the HR interviews. Once the material had been coded individually, it was thoroughly discussed to derive preliminary themes. Then each coder returned to the codes to test the preliminary themes with their own codes and to develop suggestions for final themes. Finally, the suggestions for final themes were discussed among the coders until consensus was reached (see Figure 1 for an example of the coding procedure).

### Ethics Statement

This study has been approved by the Regional Ethics Board in Stockholm, Sweden (Ref. No.: 2016/1208-31/5).

## Results

Based on the coded material, six themes were derived: (1) Expectations of the skills-development training; (2) Experiences of and prior beliefs about problem gambling; (3) A good basis (4) The difficult conversation; (5) Appreciated aspects of the training sessions; and (6) Remaining obstacles (see Table 1 for examples of quotes and codes for each theme).

**TABLE 1:** Examples of how themes and codes were derived from meaning units.

**Theme**	**Code**	**Meaningful unit**
Expectations of the skills-development training	Expectations, skills development, tools	“*What you expect is to get some kind of tool to help you deal with issues that may arise*”
Experiences of and prior beliefs about problem gambling	Gambling beliefs, gambling in the media	“*You can see more and more about gambling on the TV and on the radio, it never ends, and sometimes people should realize that it increases and that people get addicted to gambling* [*…*]”
A good foundation	Take-always, lessons learned, act early	“*One thing in particular that I learned is that we should act early, not wait or try to gather evidence.*”
The difficult conversation	Integrity, non-confrontational	“*It’s important not to have a confrontational style but rather to invite a discussion*”
Appreciated aspects of the training session	Positive to training, cases, exercises,	“*Using cases and exercises was rewarding and appreciated*”
Remaining obstacles	Unclear policy, dissemination of new knowledge	“*The policy was unclear for the employees*”

### Expectations of the Skills-Development Training

The theme “Expectations of the skills-development training” summarizes the participants’ thoughts and expectations toward the skills-development training. Most HR officers had positive expectations, while most managers had no expectations. There may be several reasons for this discrepancy. One reason may relate to the HR being more involved in the organization that agreed to participate in the study. Thus, the HR were more positive toward the intervention and had had more time to think about it. Another reason may relate to employee health perhaps being a topic of greater relevance to HR than to managers. Some of the expectations that were mentioned involved learning practical skills and getting advice on how to handle gambling-related problems, how to deal with an employee with PG, and how to feel more confident in situations related to harmful use.

*I think I wanted tools, I wanted to give the managers tools. But also, knowledge like, how big of an issue it is between different groups. Between young and old, men and women… And perhaps between different types of workplaces.* [HR officer, man]

The most frequently mentioned expectation was to get something concrete out of the training, mostly related to acquiring a skill or a tool.

*You expect to get some kind of tool to help you with this…you don’t know what to say or do.* [HR officer, woman]

Others mentioned that they wished for the adaptation of the skills-development training to the workplace in question (e.g., noticing warning signs when working at an organization with flexible working hours). Although most expectations were either positive or non-existent, there was one notable exception. This involved having negative expectations but then changing one’s mind while attending the training.

*I expected it to be something I just had to sit through, but it actually turned out very interesting.* [Manager, man]

### Experiences of and Prior Beliefs About Problem Gambling

The theme “Experiences of and prior beliefs about problem gambling” describes the participants’ own experiences and prior understanding of PG. Mostly, participants had no earlier experience of PG in their private lives or in their workplaces. However, some said that gambling at work was becoming more common. Regardless of any earlier experiences of PG or a lack thereof, participants felt that both gambling and prevention were, in general, relevant topics that they wanted to know more about. However, participants had different ideas of what was meant by “gambling.” Some participants mentioned colleagues who gambled in online casinos during work, whereas others mentioned sports betting together with colleagues.

*And that’s something I’ve noticed here. We’ve said that you shouldn’t sit with your smartphone, but there is always someone fiddling around at one of these slot sites to gamble, and that is much worse now than earlier, we can almost guarantee it; at least that’s what I see.* [Manager, man]

*I see now that we have a problem here at work […] We’ve always had a group of sports betters; probably half of the office joined it.* [Manager, woman]

Several participants steered the conversation to their general thoughts on gambling. These were mostly related to the high frequency of gambling advertising in the media, and the perceptions were all negative.

*You see more and more about gambling now on the TV and on the radio; there’s no end to it.* [Manager, woman]

### A Good Basis

The theme “A good basis” reflects the participants’ thoughts on what the program as a whole gave them. Each of the elements in this theme provides a good basis for working with PG and other types of harmful use in the workplace. Some participants found it useful just to learn that PG is an actual issue in the workplace and that they need to keep it in mind. Some considered it particularly useful to know when managers have the mandate to act on their concerns about someone having problems with gambling in the workplace. The importance of information about potential risk factors in the working environment, such as flexible working hours, and how the working environment can be modified to avoid risk factors were also mentioned as important elements in building a good foundation. Furthermore, learning about other forms of harmful use was also considered useful, while getting help in developing or strengthening workplace policy was appreciated.

*And then I think… just the awareness… that it’s not only about alcohol and drugs.* [Manager, woman]

The combination of support for policy development and implementation and skills-development during the same time period was considered appropriate. The participants mentioned that it was important to learn about the early signs of PG or other harmful use.

*To be able to pick folks, to pick out the employees at an earlier stage so that we, hopefully, can prevent those really tragic incidents.* [Manager, man]

### The Difficult Conversation

One key part of a good basis was related to the topic of “The difficult conversation,” i.e., initiating a conversation with an employee suspected of having a harmful use issue. The participants expressed increased confidence in their own ability to conduct such a “difficult conversation” as a result of the intervention. The recommendations to act on the first sign that something might affect work and not to await supporting evidence were described as important factors in reducing inhibitions about having such a conversation.

*Before the training I had probably, it had taken longer before I had told management that there was gambling in my department.* [Manager, male]

Furthermore, framing the conversation as “unfamiliar” instead of “difficult” was considered useful for the reason that no one wants to have difficult conversations, but everyone can practice becoming better at having unaccustomed ones.

### Appreciated Aspects of the Training Session

This theme directed the participants’ thoughts to what they appreciated the most about the training sessions. Among the aspects that were often recalled were the cases describing individuals with gambling problems and the consequences that followed. These cases seemed to leave participants with a sense of the importance and seriousness of the issue of PG.

*I think they showed just the tip of the iceberg. They showed how little you can see compared to everything below the surface that you cannot really see…what signals there are and how they relate to stress. Like, a stressful situation in general can have the same symptoms as if you were gambling or had another harmful use issue.* [HR officer, woman]

Furthermore, the participants also appreciated learning general statistics and facts about PG.

*They gave many examples, many cases that were interesting to hear about.* [Manager, man]

*And even if I don’t remember all of the statistics precisely, I usually get these “Aha!” experiences: “Aha! It’s that much, yes”.* [HR officer, woman]

The exercises were appreciated as they increased the level of activity and engagement among the participants.

*But we also had some of these exercises when people stand in different corners of the room, and some may think that it is dorky, but it’s always stuff like this that kick-starts the participants and makes things more visible, and that gets the conversations going between unexpected parties, for example, when the principal safety representative starts talking to a manager.* [Manager, woman]

The opportunity to practice having a conversation with an employee who might be at risk of developing PG was also considered useful.

*There were cases with different people and situations, which also was really good. And then we practiced; it was very useful to get to practice having these kinds of conversations.* [HR officer, woman]

### Remaining Obstacles

The final theme directed the participants’ thoughts to obstacles that still remained after the program as well as to issues with the program as a whole. One issue with the skills-development sessions was the lack of tailoring of the material to a particular organization. Specifically, this included organizations without fixed working hours and organizations where neuropsychiatric disorders were prevalent among employees. Another obstacle pertaining to the “difficult conversation” was centered around the dilemma of private life versus the workplace.

*If someone’s got a problem, then it’s someone who’s going through some difficult times and who might experience it as an even greater violation of privacy, compared to someone who’s being questioned without any support, so to speak. So, you must be prepared, I guess, for quite hostile situations, different types of reactions that aren’t comfortable…for the person who initiated this talk as well. So, it’s surrounded by lots of difficulties.* [Manager, man]

Obstacles related to the intervention as a whole were discovered when participants were asked about the two tools: the checklist and the template. While some participants said the checklist might come in handy, others had forgotten about getting a checklist or did not know what it was for.

*No, I don’t think I remember that, but I assume we got some material with us which I can look up if needed.* [Manager, female]

Lastly, participants feared that the lack of communication might impede further workplace changes after the intervention. It was not clear to some participants how the new policies would reach the employees. Another communication issue pertained to perceptions of policy changes: when asked about whether there was a procedure to identify and handle suspected cases of PG, the managers either did not know or thought that there was no established procedure. In contrast, the HR personnel stated that a policy update was on its way. However, some participants suspected the new policy might not be clear enough for colleagues who did not participate in the training.

*Gambling—it’s very vague. It didn’t turn out to be that specific. It’s not that up-to-date, so you can understand that. For us who’ve been through the training, that’s one thing. We understand the significance of this, but I don’t think it’s particularly clear to the staff who read it for the first time, what it actually means. So, that’s why I feel that we need to talk about it. We need to work with it. We need to make it more natural, you know, like in how we behave and talk and….* [Manager, woman]

## Discussion

In this study, participants in a workplace-oriented problem gambling prevention program were interviewed regarding their experiences. Two components were included in the intervention: policy implementation and skills-development training. Despite differences in their previous knowledge about PG, the participants generally reported being satisfied with the intervention. In the skills-development training, the case presentations and statistics were appreciated. Some of the positive consequences of the intervention that were well received were related to general knowledge about PG, getting help with policy development, and becoming more inclined to conduct the “difficult conversation.” In particular, to act on the first sign of harmful use and not to chase supporting evidence were important insights, as well as believing in one’s ability to conduct the conversation.

The appreciated aspects of the skills-development sessions provide some ideas about what similar educational measures could include in the future to appeal to the participants. A common feedback involved the suggestion to tailor the intervention and cases to the specific type of workplace the participants currently work in. This is in line with earlier research of employers views on the promotion of WHPPs (e.g., Pescud et al., 2015). Examples include organizations with employees with disabilities and organizations with highly flexible and individualized working conditions. Although, to our knowledge, no research has compared tailored and non-tailored interventions for PG workplace programs, meta-analytical evidence suggests that tailoring enhances effectiveness on the individual level (Noar et al., 2007). Another suggestion was to help participants disseminate the information in the organization after the intervention, minimizing the risk of the lessons learned from the intervention gradually being forgotten.

Most of the interviews—and thus the results of the analysis—were centered on the skills-development training, while fewer concerned the policy implementation. The lack of data related to policy implementation is unfortunate, given the potential importance of workplace policies for dealing with problem gambling. For instance, Pidd et al. (2006) found that the presence of workplace policies for harmful use in general was associated with lower use of alcohol and cannabis. One possible reason for the lack of reported experiences of policy implementation is that participants were recruited at the skills-development training, which means that their level of involvement with policy varies. However, the fact that policy implementation seems to have passed rather unnoticed among managers may also indicate that, similar to the skills-development training, a strategy to disseminate the new information among managers and HR officers needs to be more clearly defined. Considering the findings of McCleary et al. (2017), who noted that only half as many employees as managers in the United States reported having access to a WHPP, the dissemination strategy needs to include employees as well. In contrast to the cultural model to prevent and treat alcohol-related problems (Pidd and Roche, 2008), the current intervention included no employee education to raise awareness of the new policy, which is considered a limitation. Although the current intervention was aimed only at managers, future interventions should incorporate efforts to raise awareness among other employees as well.

Although participants reported being satisfied with getting help in writing and implementing policies, some participants were dissatisfied with the phrasing of the new policies, stating, for example, that the text may not be clear enough for those who have not attended the skills-development training. Thus, following up on the feasibility of the written policies should be a crucial part of implementing policy. Besides following up on the policy content, it is necessary to follow up on policy compliance and obstacles to compliance. As noted by Antin et al. (2010), social relationships and any tendency to avoid confrontation may complicate the policy use.

The intervention also included distributing an action checklist to managers and a policy template to HR officers. The participants had difficulties recalling the documents, which suggests that the documents might not be used as intended. Further research should investigate if, and under what circumstances, this kind of supplementary material is effective.

The limitations of this study include interviewing a non-random sample of participants, i.e., participants who actively signed up for interviewing during the skills-development sessions. Had the sample been randomized, it is possible that other aspects might have been discussed. Furthermore and contrary to recommendations for qualitative content analysis (Drisko and Maschi, 2015), the sample was not iteratively increased until no further content appeared in the interviews. Instead, the sample size was based on the number of available participants. However, the data generated from interviewing the twenty-three participants was perceived as comprehensive by both coders and interviewers (Supplementary Material). Having multiple interviewers may also have impacted the result, but the interview guide used by the interviewers allowed all participants to elaborate on the same topics.

Another limitation involves the skills-development training not focusing exclusively on PG; other types of harmful use were mentioned briefly as well. The organization delivering the training provided two key arguments to justify including other types of harmful use. First, gambling can be considered easier to understand when it is related to more well-known types of harmful use (e.g., while you can feel the smell of alcohol of someone who has been drinking, it is impossible to smell a night of gambling). The other argument relates to recruitment of participants; participants are more likely to attend if the training not only involves gambling. These arguments are based on previous experience only. Thus, without any empirical studies, the validity of these arguments remains unknown. Yet, with this organization and other organizations applying a similar procedure in any future use of the intervention, this increases the ecological validity of the procedure and our findings.

Furthermore, asking participants after the intervention what they were thinking before the intervention may run the risk of recall bias. This may have led to the conflating of actual expectations with critique. For example, the same participant who mentioned that the skills-development training would have benefited from being customized to the organization also mentioned this as an expectation. This could have been mitigated by asking about expectations before the intervention and then following this up during a post-training interview. However, the expectations and the critique are welcome contributions nonetheless.

Finally, the intervention was delivered by different consultants, which may have yielded different effects. With all consultants using the same material to deliver the intervention, participants should have received the same information. Still, individual consultant characteristics, such as likeability, may influence participants’ perceptions of the intervention.

## Conclusion

The findings of this study provide ideas about implementing this specific intervention in particular and implementing workplace interventions in general. Regarding the intervention examined in this study, both strengths and weaknesses were found. The creation of a feasible skills-development training program with relevant components can be considered a strength. Weaknesses include not tailoring the skills-development to the organization and including components that were not used by the participants (i.e., the template and the checklist). Regarding workplace interventions in general, the insights of this study mainly concern what intervention components were appreciated by the participants. These included getting the support of consultants in the implementation process as well as following up on the results over time to minimize the risk of the potential changes inspired by the training gradually fading away.

## Ethics Statement

The protocol was approved by the Regional Ethics Board in Stockholm, Sweden (Ref. No.: 2016/1208-31/5). All subjects gave written informed consent in accordance with the Declaration of Helsinki.

## Author Contributions

JR, PC, and EI coordinated the study. AR and PC supervised the students who performed the interviews. JR, EI, and AR conducted the analyses. JR and EI wrote the first draft of the manuscript. All authors were involved in finalizing the manuscript.

## Conflict of Interest Statement

The authors declare that the research was conducted in the absence of any commercial or financial relationships that could be construed as a potential conflict of interest.

## References

[B1] Alna J. (2015). Mall För Arbete Med Spel Och Spelproblem På Arbetsplatsen [Template For Working With Gambling And Problem Gambling in The Workplace. Available at: https://www.alna.se/sites/default/files/mall_for_arbete_med_spel_och_spelproblem_pa_arbetsplatsen.pdf (accessed March 18, 2018).

[B2] Antin T. M. J. Moore R. S. Lee J. P. Satterlund T. D. (2010). Law in practice: obstacles to a smokefree workplace policy in bars serving asian Patrons. *J. Immigr. Minor. Health* 12 221–227. 10.1007/s10903-008-9174-y 18712482 PMC2918413

[B3] Binde P. (2016a). Gambling-related embezzlement in the workplace: a qualitative study. *Int. Gambl. Stud.* 16 391–407. 10.1080/14459795.2016.1214165

[B4] Binde P. (2016b). Preventing and responding to gambling-related harm and crime in the workplace. *Nord. Stud. Alcohol Drug* 33 247–265. 10.1515/nsad-2016-0020

[B5] Browne M. Rockloff M. J. (2018). Prevalence of gambling-related harm provides evidence for the prevention paradox. *J. Behav. Addict.* 7 410–422. 10.1556/2006.7.2018.41 29788761 PMC6174604

[B6] Bruening R. A. Strazza K. Nocera M. Peek-Asa C. Casteel C. (2015). Understanding small business engagement in workplace violence prevention programs. *Am. J. Health Promot.* 30 e83–e91. 10.4278/ajhp.140221-QUAL-80 25806571

[B7] Buchberger B. Heymann R. Huppertz H. Friepörtner K. Pomorin N. Wasem J. (2011). The effectiveness of interventions in workplace health promotion as to maintain the working capacity of health care personal. *GMS Health Technol. Assess.* 7:Doc06. 10.3205/hta000097 22031811 PMC3198117

[B8] Canale N. Vieno A. Griffiths M. D. (2016). The extent and distribution of gambling-related harms and the prevention paradox in a british population survey. *J. Behav. Addict.* 5 1–9. 10.1556/2006.5.2016.023 27156382 PMC5387771

[B9] Casey L. M. Oei T. P. S. Raylu N. Horrigan K. Day J. Ireland M. (2017). Internet-based delivery of cognitive behaviour therapy compared to monitoring, feedback and support for problem gambling: a randomised controlled trial. *J. Gambl. Stud.* 33 993–1010. 10.1007/s10899-016-9666-y 28124288

[B10] Delfabbro P. King D. (2017). Prevention paradox logic and problem gambling: does low-risk gambling impose a greater burden of harm than high-risk gambling? *J. Behav. Addict.* 6 163–167. 10.1556/2006.6.2017.022 28425779 PMC5520119

[B11] Drisko J. Maschi T. (2015). *Content Analysis.* Oxford: Oxford University Press

[B12] Durlak J. A. DuPre E. P. (2008). Implementation matters: a review of research on the influence of implementation on program outcomes and the factors affecting implementation. *Am. J. Commun. Psychol.* 41 327–350. 10.1007/s10464-008-9165-0 18322790

[B13] Elo S. Kyngäs H. (2008). The qualitative content analysis process. *J. Adv. Nurs.* 62 107–115. 10.1111/j.1365-2648.2007.04569.x 18352969

[B14] Goetzel R. Z. Henke R. M. Tabrizi M. Pelletier K. R. Loeppke R. Ballard D. W. (2014). Do workplace health promotion (wellness) programs work? *J. Occup. Environ. Med.* 56 927–934. 10.1097/JOM.0000000000000276 25153303

[B15] Griffiths M. (2009). Managing internet gambling in the workplace. *J. Workplace Learn.* 21 658–670. 10.1108/13665620910996197

[B16] Hawley C. E. Glenn M. K. Diaz S. (2007). Problem gambling in the workplace, characteristics of employees seeking help. *Work* 29 331–340. 18057573

[B17] Hendriksen I. J. M. Snoijer M. De Kok B. P. H. Van Vilsteren J. Hofstetter H. (2016). Effectiveness of a multilevel workplace health promotion program on vitality, health, and work-related outcomes. *J. Occup. Environ. Med.* 58 575–583. 10.1097/JOM.0000000000000747 27136605 PMC4883645

[B18] Justesen J. B. Eskerod P. Christensen J. R. Sjøgaard G. (2017). Implementing workplace health promotion – role of middle managers. *Int. J. Workplace Health Manag.* 10 164–178. 10.1016/j.jamda.2014.08.018 25306289

[B19] Kilpatrick M. Sanderson K. Blizzard L. Nelson M. Frendin S. Teale B. (2014). Workplace health promotion: what public-sector employees want, need, and are ready to change. *J. Occup. Environ. Med.* 56 645–651. 10.1097/JOM.0000000000000161 24854258

[B20] Langham E. Thorne H. Browne M. Donaldson P. Rose J. Rockloff M. (2016). Understanding gambling related harm: a proposed definition, conceptual framework, and taxonomy of harms. *BMC Public Health* 16:80. 10.1186/s12889-016-2747-0 26818137 PMC4728872

[B21] Larsson R. Akerlind I. Sandmark H. (2016). Managing workplace health promotion in municipal organizations: the perspective of senior managers. *Work* 53 485–498. 10.3233/WOR-152177 26519015

[B22] Magnavita N. Capitanelli I. Falvo R. La Milia D. I. Borghini A. Moscato U. (2017). Workplace health promotion programs in different areas of Europe. *Epidemiol. Biostat. Public Health* 14 1–8. 10.2427/12439

[B23] Magnusson K. Nilsson A. Hellner Gumpert C. Andersson G. Carlbring P. (2015). Internet-delivered cognitive-behavioural therapy for concerned significant others of people with problem gambling: study protocol for a randomised wait-list controlled trial. *BMJ Open* 5:e008724. 10.1136/bmjopen-2015-008724 26656017 PMC4680021

[B24] Marshall D. (2009). Gambling as a public health issue: the critical role of the local environment. *J. Gambl. Issues* 23 66–80. 10.4309/jgi.2009.23.4

[B25] McCleary K. Goetzel R. Z. Roemer E. C. Berko J. Kent K. De La Torre H. (2017). Employer and employee opinions about workplace health promotion (wellness) programs: results of the 2015 harris poll nielsen survey. *J. Occup. Environ. Med.* 59 256–263. 10.1097/JOM.0000000000000946 28267097

[B26] Noar S. M. Benac C. N. Harris M. S. (2007). Does tailoring matter? Meta-analytic review of tailored print health behavior change interventions. *Psychol. Bull.* 133 673–693. 10.1037/0033-2909.133.4.673 17592961

[B27] Nyqvist M. (2015). *Checklista för Chefer Vid Spelproblem [Problem Gambling Checklist for Managers.*

[B28] Pescud M. Teal R. Shilton T. Slevin T. Ledger M. Waterworth P. (2015). Employers’ views on the promotion of workplace health and wellbeing: a qualitative study. *BMC Public Health* 15:642. 10.1186/s12889-015-2029-2 26162910 PMC4499225

[B29] Pidd K. Boeckmann R. Morris M. (2006). Adolescents in transition: the role of workplace alcohol and other drug policies as a prevention strategy. *Drugs Educ. Prevent. Policy* 13 353–365. 10.1080/09687630600700137

[B30] Pidd K. Kostadinov V. Roche A. (2016). Do workplace policies work? *Int. J. Drug Policy* 28 48–54. 10.1016/j.drugpo.2015.08.017 26410610

[B31] Pidd K. Roche A. (2008). “Changing workplace cultures: an integrated model for the prevention and treatment of alcohol-related problems,” in *Drugs and Public Health: Australian Perspectives on Policy and Practice*, eds Moore D. Dietze P. (Melbourne: Oxford University Press).

[B32] Public Health Agency of Sweden (2016a). *Gambling and Gambling Problems in Sweden 2008–2010 – Swedish Longitudinal Gambling Study, Swelogs. Findings From Wave one and wave two.* Available at: https://www.folkhalsomyndigheten.se/contentassets/fe1d3ba6e62a40158a8188bb3b83b9c5/gambling-gambling-problems-sweden-2008-2010-16013.pdf (accessed February 3, 2018).

[B33] Public Health Agency of Sweden (2016b). *Tabellsammanställning för Swelogs Prevalensstudier 2015 [Table Compilation for Swelogs Prevalence Studies.* (Sweden: Public Health Agency of Sweden)

[B34] Rafi J. Ivanova E. Rozental A. Carlbring P. (2017). Effects of a workplace prevention programme for problem gambling: study protocol for a cluster randomised controlled trial. *BMJ Open* 7 1–8. 10.1136/bmjopen-2017-015963 28951403 PMC5623572

[B35] Risica P. M. Gorham G. Dionne L. Nardi W. Ng D. Middler R. (2018). A multi-level intervention in worksites to increase fruit and vegetable access and intake: rationale, design and methods of the ‘Good to Go’ cluster randomized trial. *Contemp. Clin. Trials* 65 87–98. 10.1016/j.cct.2017.12.002 29242108 PMC5912165

[B36] Rodda S. Lubman D. (2012). Ready to change: a scheduled telephone counselling programme for problem gambling. *Aust. Psychiatry* 20 338–342. 10.1177/1039856212449671 22767931

[B37] Ryu H. Jung J. Cho J. Chin D. L. (2017). Program development and effectiveness of workplace health promotion program for preventing metabolic syndrome among office workers. *Int. J. Environ. Res. Public Health* 14:E878. 10.3390/ijerph14080878 28777320 PMC5580582

[B38] Spurling M. C. Vinson D. C. (2005). Alcohol-related injuries: evidence for the prevention paradox. *Ann. Fam. Med.* 3 47–52. 10.1370/afm.243 15671190 PMC1466795

[B39] Svensson J. Romild U. Shepherdson E. (2013). The concerned significant others of people with gambling problems in a national representative sample in Sweden - a 1 year follow-up study. *BMC Public Health* 13:1087. 10.1186/1471-2458-13-1087 24261955 PMC3870974

[B40] Vaismoradi M. Turunen H. Bondas T. (2013). Content analysis and thematic analysis: implications for conducting a qualitative descriptive study. *Nurs. Health Sci.* 15 398–405. 10.1111/nhs.12048 23480423

[B41] Vera E. M. (2013). “The Oxford handbook of prevention in counseling psychology”,” in *The Oxford Handbook of Prevention in Counseling Psychology*, ed. Vera E. M. (Oxford: Oxford University Press), 10.1093/oxfordhb/9780195396423.001.0001

[B42] Webb G. Shakeshaft A. Sanson-Fisher R. Havard A. (2009). A systematic review of work-place interventions for alcohol-related problems. *Addiction* 104 365–377. 10.1111/j.1360-0443.2008.02472.x 19207344

[B43] Wild T. C. Macdonald S. Csiernik R. Durand P. Rylett M. Wild T. C. (2006). Prevalence and factors related to canadian workplace health programs. *Can. J. Public Health* 97 121–125. 10.2307/41994698 16619999 PMC6975878

[B44] Wiman V. Lydell M. Nyholm M. (2016). Views of the workplace as a health promotion arena among managers of small companies. *Health Educ. J.* 75 950–960. 10.1177/0017896916643355

[B45] Wood R. T. Williams R. J. (2007). Problem gambling on the internet: implications for internet gambling policy in North America. *New Media Soc.* 9 520–542. 10.1177/1461444807076987

[B46] Wynne H. Ferris J. (2001). *The Canadian Problem Gambling Index: Final Report.* Ottawa: Canadian Centre on Substance Abuse.

