# Supplementary material for: Participants’ Experiences of a Workplace-Oriented Problem Gambling Prevention Program for Managers and HR Officers: A Qualitative Study
Source: Front Psychol. 2019 Jul 3;10:1494. doi: 10.3389/fpsyg.2019.01494 (PMC6616087; doi:10.3389/fpsyg.2019.01494)
Supplement: Supplementary file 1 [file Table_1.DOCX]

1. **Introduction**
   1. Welcome and thank you for participating
   2. Inform of the time (about 45 minutes) and that the conversation is being recorded. Inform about confidentiality. Ask for a signed written consent if they agree to participate.
   3. Explain the interview outline: that you will use the interview guide to direct the conversation, but that you may discuss freely.
   4. Ask if there are any questions before you start.
2. **Opening questions (HR and managers)**
   1. Why did you decide to participate in the skills-development training?
   2. What was your experience of problem gambling in the workplace prior to the skills-development training?
   3. What was your experience of harmful use (e.g. alcohol) in the workplace prior to the skills-development training?
   4. Prior to the skills-development training, had there been any earlier work with gambling and problem gambling at your workplace?
3. **The template (HR only)**
   1. Why did your organization decide to participate in the prevention program?
   2. Describe the process of implementing/renewing the policy regarding problem gambling and other harmful use in your organization.
   3. Describe how your organization have used the “template for working with gambling and problem gambling in the workplace”.
   4. Describe your impression of the template. Also ask about useability, intelligibility.
   5. Has the policy-work affected the workplace?
      1. If yes:
         1. How did you notice it?
         2. Have the employees noticed anything?
   6. The purpose of the template has been to start working with problem gambling at the workplace in a structured manner. In your opinion, how well is the template corresponding to its purpose?
   7. What do you like about the template?
   8. What do you not like about the template?
4. **The skills-development training (HR and managers)**
   1. What were your expectations of the skills-development training?
      1. How did the skills-development training correspond to your expectations?
   2. Describe the skills-development training sessions.
      1. What did they include?
      2. What are your take-aways?
   3. How have the skills-development training affected your workplace?
   4. What possibilities and difficulties do you see with what you have learned?
   5. What did you like about the skills-development training?
   6. What did you not like?
   7. Would you recommend the skills-development training to other organizations?
5. **The checklist (managers only)**
   1. During the skills-development training you were presented with the “Problem gambling checklist for managers”, what do you think is the purpose with it?
   2. The purpose of the checklist is to be a supporting guide to encourage early interventions and conversations. How well do you think the checklist corresponds to its purpose?
   3. What are your thoughts about the checklist?
   4. To what extent have you used the checklist?
   5. In what way do you think you will use the checklist?
   6. What do you like about the checklist?
   7. What do you not like about the checklist?
6. **Other: (HR and managers)**
   1. How has the number of conducted actions regarding problem gambling or other harmful use been affected after the prevention program?
   2. Is there anything else you want to disclose about the project and its activities in your organization?
